# Less missing values—evaluation of proteomics workflows for the quantification of (small) proteins

**DOI:** 10.1093/femsml/uqag002

**Published:** 2026-01-10

**Authors:** Jürgen Bartel, Vaikhari Kale, Dennis Joshua Pyper, Harald Schwalbe, Sandra Maaß

**Affiliations:** Department of Microbial Proteomics, Institute of Microbiology, University of Greifswald, 17489 Greifswald, Germany; Department of Microbial Proteomics, Institute of Microbiology, University of Greifswald, 17489 Greifswald, Germany; Center of Biomolecular Magnetic Resonance (BMRZ), Institute for Organic Chemistry and Chemical Biology, Goethe University Frankfurt, 60438 Frankfurt am Main, Germany; Center of Biomolecular Magnetic Resonance (BMRZ), Institute for Organic Chemistry and Chemical Biology, Goethe University Frankfurt, 60438 Frankfurt am Main, Germany; Department of Microbial Proteomics, Institute of Microbiology, University of Greifswald, 17489 Greifswald, Germany

**Keywords:** mass spectrometry, peptidomics, low molecular weight proteome, *Clostridioides difficile*, SEPs, sProteins, database search, spectral library

## Abstract

Quantitative information on protein abundance is crucial to understand biological processes and is therefore frequently gathered in proteomic studies. However, the quality of a quantitative proteomic dataset is greatly affected by the number of missing values, which need to be minimized to produce robust and meaningful data. In this context, small proteins (≤100 amino acids) pose specific analytical challenges, which hinder their efficient identification and quantitative characterization in complex proteomes. In this study, methods for sample preparation and MS-data processing are systematically evaluated for their contribution to identification and quantification of small proteins of *Clostridioides difficile* 630 Δ*erm*. Results show that small protein enrichment can enhance the number of identified and quantified proteins also for low abundant small proteins. Through application of spectral libraries for identification of MS spectra the number of robustly quantified proteins is increased and a lower limit of their detection is reached. Additionally, the dataset presented here is currently the most comprehensive protein repository for *C. difficile* covering 84.7% of the predicted proteome and 61.4% of all predicted small proteins of this important pathogen.

## Teaser

This study systematically evaluates methods for improving the identification and quantification of small proteins using bottom-up mass spectrometry, thereby providing the most comprehensive dataset of proteins from the important pathogen *Clostridioides difficile*.

## Introduction

Investigation of proteins in living systems continues to yield valuable insights into various biological processes across all domains of life. About 15 years ago, the primary objective of most proteomic experiments was the identification of expressed proteins using mass spectrometry (MS) to provide a qualitative understanding of protein composition and protein–protein interactions. However, to capture most physiological changes resulting from perturbations in a biological system, it is crucial to acquire quantitative information on protein abundance. Meanwhile, quantitative measurements form the core of nearly every proteomic study and have become routine in laboratories worldwide. However, there are specific challenges associated with the accurate quantitative description of proteomes. In commonly used bottom-up approaches, proteins are digested by a specific protease and the resulting peptides are analysed by liquid chromatography–tandem mass spectrometry (LC–MS/MS) (Zhang et al. 2013). In most cases, successful protein identification requires only detection of a sufficient number of associated peptides in at least one of the samples. In contrast, valid quantitative values under at least two different conditions need to be available to compare protein abundances. In order to statistically validate the quantitative information, these quantitative values need to be present in multiple biological replicates. Hence, the quality of a quantitative proteomic dataset is greatly affected by the number of missing values contained. Missing values appear due to different reasons. A protein might simply not be expressed in a given condition or its abundance might be below the limit of detection for the given experimental setup. Moreover, the stochastic nature of data-dependent MS measurements and applied filters for protein quantification (e.g. the necessity of identifying at least two unique peptides per protein) may contribute to the extent of missing values in a dataset. Although missing values can be handled differentially during data processing (e.g. by removing corresponding protein hits, by using only valid values, or by imputation of missing values), this data processing can have a significant impact on the results of the analyses. Therefore, it is desirable to generate a robust quantitative dataset with the least possible number of missing values.

Proteins with a very small size (here defined as “small proteins” with a length ≤100 amino acids) pose specific analytical challenges. They can be easily lost during sample preparation and, signals from larger proteins often mask them during ionization, separation, and detection in MS. These issues hinder their efficient identification and quantitative characterization. This led to the underrepresentation of small proteins in current proteomic studies although it has been shown that these proteins are often involved in important physiological functions, such as metabolism (Krauspe et al. 2021, Alvarenga-Lucius et al. 2023), signaling (Weel-Sneve et al. 2013, Voichek et al. 2020), virulence (Arnison et al. 2013, Venturini et al. 2020, Edelmann and Berghoff 2022), multiresistance (Melior et al. 2020), or regulation of enzyme activity (Yin et al. 2019, Gutt et al. 2021, Song et al. 2022). To meet the analytical challenges of small proteins, which are most often related to their short sequence and low abundance in a total protein background, different strategies have been proposed to successfully enrich small proteins from various samples. These approaches include membrane or gel filtration (Hu et al. 2007, Klein et al. 2007, Petruschke et al. 2021), two-dimensional LC-based prefractionation (Cassidy et al. 2021), differential precipitation (Cassidy et al. 2019), as well as solid phase extraction (Bartel et al. 2020). As small proteins vary in their physicochemical properties, it is improbable that a single method can effectively enrich all small proteins in an organism. In fact, a study comparing various sample preparation workflows and enrichment methods for small proteins found that the most comprehensive set of identified small proteins is achieved by combining different approaches (Petruschke et al. 2021). Beside sample enrichment, the utilization of modified digestion protocols can aid in the discovery of small proteins (Bartel et al. 2020, Müller et al. 2010, Swaney et al. 2010, Meyer et al. 2014, Osbak et al. 2016, Giansanti et al. 2016).

To enhance the number of identified small proteins further, the simultaneous utilization of multiple search engines has proven advantageous (Bartel et al. 2020, Müller et al. 2010, Wen et al. 2015) which is likely attributed to variations in spectrum preprocessing and scoring functions implemented in different search algorithms, leading to slightly different sets of reported peptides (Searle et al. 2008). MS-based identification of peptides is achieved by matching fragment ion spectra to peptide sequences and builds the base for protein identification, quantification, and thus biological interpretation (Mallick and Kuster 2010). Currently, the standard approach is database searching (Sinitcyn et al. 2018), where masses observed in actually acquired fragment spectra are compared to masses of theoretical spectra using computational methods. In this process, intensity information is disregarded. One approach to leverage the intensity information is to assemble spectral libraries from previous peptide identification data. These experimental spectral libraries are hypothesis-free regarding the content of the spectra but consider qualitative and quantitative characteristics of fragment spectra (Schubert et al. 2015, Griss 2016, Shao and Lam 2017), thus providing numerous advantages in terms of peptide validation and robust identification in extensive datasets. As experimental spectral libraries have the capability to accommodate nonstandard peaks, such as neutral losses, their application usually results in a higher number of identified proteins with less missing values in quantitative datasets (Junker et al. 2018a, Hentschker et al. 2020). However, generation of experimental spectral libraries requires labor and time intensive MS experiments as any peptide in the sample that lacks a corresponding library spectrum would be lost in the analysis. Alternatively, deep learning methods can meanwhile predict fragment spectra based on the amino acid sequence of any peptide (Gessulat et al. 2019, Gabriels et al. 2019, Cox 2023). Indeed, it was shown already that the additional information contained in predicted spectral libraries increase the number and correctness of peptide identification in standard proteome experiments (Silva et al. 2019, Gessulat et al. 2019, Tiwary et al. 2019). However, most prediction tools are currently not trained on nontryptic data or longer peptides, which might be obtained from semispecific cleavage or due to the very small size of a protein, making their use for small protein discovery and quantification nonoptimal. Consequently, we are not aware of any published study investigating the benefit of predicted spectral libraries for (small) protein quantification.

Over the last two decades, the anaerobic bacterium *Clostridioides difficile* has emerged as the primary cause of antibiotic-associated diarrhea (Smits et al. 2016). The treatment of *C. difficile* infections (CDI) is confronted by high rates of recurrent infections (Johnson 2009) and escalating numbers of multidrug-resistant clinical isolates (Peng et al. 2017). The clinical difficulties associated with CDI have spurred considerable efforts to understand how *C. difficile* modulates its virulence. Currently, there is a comprehensive body of literature (Table S1) with evidence for translation of up to 2385 proteins (and up to 119 small proteins) in a single study (Trautwein-Schult et al. 2018). However, there is still no evidence yet for the active translation of 1077 proteins (28.5% of the predicted proteome) (Dannheim et al. 2017) including 203 small proteins (58% of the 350 yet annotated small proteins).

Besides their identification, the quantitative comparison of small protein abundances poses an even greater challenge as quantitative values are often based on only a single peptide, preventing any statistical analysis within the protein. Consequently, systemic investigations on robust quantification of small proteins by nontargeted MS are still lacking. In this study, we systematically evaluated sample processing protocols and the performance of sequence databases (SD), experimental spectral libraries and predicted spectral libraries for the identification and quantification of proteins with special emphasis on small proteins (≤100 amino acids) in *C. difficile*. Finally, with the extensive dataset generated in this study, we enhanced the number of high-confident MS evidence for *C. difficile* proteins and report the extended proteome of this important pathogen.

## Materials and methods

### Bacterial strain and cultivation

*Clostridioides difficile* 630 Δ*erm* (Hussain et al. 2005) was grown either in brain heart infusion (BHI; Oxoid, Basingstoke, UK) or chemically defined medium (CDMM) (Neumann-Schaal et al. 2015) in an anaerobic chamber (Don Whitley Scientific Ltd., Bingley, UK) at 37°C to exponential or early stationary phase. For this purpose, spores were germinated in BHI containing 0.1% (w/v) taurocholate for at least 24 h and precultures were inoculated by adding 0.1% (v/v) germinated spore solution to cold BHI or CDMM, respectively. After ~16 h, precultures were diluted to an OD_600nm_ of 0.05 in the corresponding medium. When the cultures reached the desired growth phase, samples were obtained by centrifugation at 10 000 × *g* and 4°C for 5 min, cell pellets were washed twice with ice-cold Tris–HCl buffer (50 mM, pH 8.0), and stored at −20°C. Growth experiments were carried out in three independent replicates.

### Sample preparation

For protein extraction, cell pellets were resuspended in Tris–HCl buffer and mechanically disrupted with 0.1 mm diameter glass beads for five homogenization cycles (6.5 m/s^2^) of 30 s each in a FastPrep-24 5G instrument (MP Biomedicals, Irvine, USA). Samples were cooled on ice for 5 min between the cycles. Cell debris and glass beads were removed by a mild centrifugation step at 8000 × *g* and 4°C for 5 min, which ensured minimal loss of membrane-associated small proteins. Protein concentration was determined by Bradford assay using bovine serum albumin as an external calibrant. Aliquots of 500 µg protein amount were used for small protein enrichment by solid phase extraction columns as previously described (Bartel et al. 2020). The enriched small protein fraction and total protein samples were digested with trypsin using S-Trap spin columns (Protifi, Huntington, USA) as described in Kroniger et al. (2022) except that Tris(2-carboxyethyl)phosphine instead of dithiothreitol was used as the reducing agent. Generated peptides were purified with 100 µl Pierce C18 Tips and their concentration was determined by Pierce Quantitative Fluorometric Peptide Assay (both Thermo Fisher Scientific, Waltham, USA) according to the manufacturer’s protocols. Prior to MS measurement, retention time calibration peptides (iRT, Biognosys, Schlieren, Switzerland) were added to the samples and constant peptide amounts were injected for total protein or small protein-enriched samples within each experiment.

In order to enhance the number and quality of spectra obtained in the experimental spectral libraries, 175 synthetic tryptic peptides of 55 *C. difficile* proteins (Table S2) were purchased from JPT Peptide Technologies GmbH (Berlin, Germany) in 96-well plate format. 250 µg of each peptide was pooled and the resulting stock solution was diluted in 10% (v/v) acetonitrile and 0.1% (v/v) acetic acid to reach a final concentration of 1 ng/µl for each peptide. Finally, 10 µl of a subsequent 1:10 dilution in 0.1% (v/v) acetic acid were injected for each LC–MS/MS run resulting in 1 ng of each peptide on column.

To determine the limits of small protein quantification, protein extraction was performed from one biological replicate grown in BHI to the early stationary phase as described above. After protein concentration determination, samples were spiked with a mixture of six purified small proteins from *Haloferax volcanii* H119 (see Supplemental material) in technical triplicates. Spike-in concentrations were selected to cover the range of low- and medium-abundant proteins and were: 0.5, 1.0, 5.0, 10, 50, 100, 250, and 500 pg/µg *C. difficile* proteome. After adding the *Haloferax* proteins, sample aliquots were stored immediately at −80°C and processed replicate-wise on different days as described above for the other samples. For spectral library generation, a mixture of 1 µg of each of the six purified *Haloferax* proteins was digested as described above.

### MS

LC–MS/MS analyses were performed with an EASY-nLC II liquid chromatography system coupled to an LTQ Orbitrap Velos (Thermo Fisher Scientific). Peptides were loaded on a self-packed analytical column (OD 360 µm, ID 100 µm, and length 20 cm) filled with 3 µm diameter C18 particles (Dr. Maisch HPLC GmbH). Peptides were eluted using a binary nonlinear gradient of 5%–99% acetonitrile in 0.1% acetic acid over 157 min at a flow rate of 300 nl/min, and subjected to electrospray ionization-based MS. A full scan in the Orbitrap with a resolution of 60 000 was followed by CID of the twenty most abundant precursor ions. MS/MS experiments were acquired in the linear ion trap.

LC–MS/MS analyses of synthetic peptides were performed as described above, except that the peptides were eluted by a shorter 90-min binary nonlinear gradient of 5%–99% acetonitrile in 0.1% acetic acid.

### SD and spectral libraries

Spectra from MS raw files were identified either by searching them against a SD or against spectral libraries. The SD contained 3781 entries for *C. difficile* 630 Δ*erm* (Dannheim et al. 2017). Different spectral libraries were applied, namely an experimental spectral library (ExSpLib), a spectral library generated by machine learning (MaLeSpLib) and a merged spectral library (merged SpLib) where spectra predicted by machine learning were added for those peptides, which were not already contained in the ExSpLib.

The MS raw files used for the generation of the ExSpLib were obtained from previously published proteomics studies or were generated in this work. An overview on used input MS data can be found in Table S2. Data for generation of the ExSpLib were search with MSGF-Plus as this algorithm resulted in highest numbers of PSMs for CID spectra (Bartel et al. 2020). The same SD for *C. difficile* 630 Δ*erm* (Dannheim et al. 2017) as described above but with added common laboratory contaminations and reverse entries was applied. Trypsin was specified as protease thereby assuming strictly tryptic peptides for total protein samples and a semitryptic digest for small protein enriched samples. Oxidation on methionine and carbamidomethylation on cysteine residues were selected as variable modification to consider that not all samples used for ExSpLib generation were reduced and alkylated during their preparation. Spectral library creation was performed according to Schubert et al. (2015) with slight modifications. Briefly, spectra and their particular identifications were linked together and hits were combined to interact.pep.XML files. Afterwards the PeptideProphet algorithm (Keller et al. 2002) was applied to adjust peptide identification probabilities. The following parameters were applied: minimum peptide sequence length of seven amino acids, use accurate mass binning, using mass errors in ppm, inclusion of decoy hits to pin down the negative distribution, exclusion of charge states 1+ and higher than 4+. Identifying spectra were filtered for a false discovery rate (FDR) of 0.01 on the level of peptide–spectrum matches. The resulting spectra were then imported into a spectral library. Spectra corresponding to the same identified peptide ion were merged to generate a representative consensus spectrum for a particular peptide species. The ExSpLib was subsequently extended with the same number of spectra as decoy hits generated by a random mass shift of the precursor and shuffling the peptide sequence (Yen et al. 2011).

Machine learning spectral libraries (MaLeSpLib) were generated based on the abovementioned SD for *C. difficile* 630 Δ*erm* (Dannheim et al. 2017) supplemented with common laboratory contaminants. All protein entries in the database file were *in silico* digested with trypsin using the generate-peptides function of the crux toolkit (McIlwain et al. 2014). For digestion, full-digest was assumed and peptides were filtered to contain up to two missed cleavage sites, a length between 6 and 55 amino acids, a minimal mass of 400 Da and a maximal mass of 10 000 Da. For protein N-termini both variants, with and without the initiating methionine, were considered. Using in-house Python scripts, the resulting peptide list was filtered to contain unique peptides only and converted to the input format of the Prosit spectrum prediction tool (Gessulat et al. 2019). According to Prosit’s prediction rules, all cysteine residues were assumed to be carbamidomethylated and peptide variants with up to three oxidized methionine residues were added if applicable. Doubly, triply, and quadruply charged precursor variants were included if the precursor mass-to-charge ratio was within the range of 200–2000 Th. Raw spectral libraries in the .msp format were created using an in-house instance of Koina and spectra were predicted with the Prosit-CID2020 model. The raw spectral libraries were imported into SpectraST (Lam et al. 2007), spectra were formally converted into consensus spectra and a shuffled decoy variant was added for each precursor. For decoy-to-protein mapping, a decoy.fasta file was generated containing reversed protein entries concatenated with the decoy peptides separated by a “KRKR” spacer sequence.

The final ExSpLib and MaLeSplib were used for all datasets in this study except for those acquired to determine the limit of small protein quantification in data-independent acquisition (DIA) mode, as these were generated after higher-energy collision-induced dissociation. The ExSpLib contained 102 234 spectra representing 66 192 peptide sequences, which include information for 2345 *C. difficile* proteins. The MaLeSpLib contained 2 294 652 spectra from 521 062 peptides of 3690 *C. difficile* proteins.

To construct the merged spectral library (merged SpLib), spectra predicted by machine learning were added for those peptide ions which were not already present in the ExSpLib by combining both spectral libraries without their decoy entries. After generating consensus spectra, shuffled decoy entries were generated as described above, mapped to the forward protein sequences and added to the merged SpLib. The final merged SpLib contained 2 309 744 spectra representing 532 129 peptide sequences of 3700 *C. difficile* proteins.

### Data processing

In order to identify spectra with the help of a classical SD, the widely applied software MaxQuant (v 2.0.3.0) was used. MS raw data were searched against a *C. difficile* 630 Δ*erm* database (Dannheim et al. 2017) with 3781 entries. Common laboratory contaminants and reverse entries were added by MaxQuant. MaxQuant was used with the following parameters: primary digest reagent, trypsin, acetylation N, K (+42.0106) and oxidation M (+15.9949) as variable modifications. Results were filtered for a 1% FDR on spectrum, peptide, and protein levels. Match between runs with default parameters was enabled. In this study, the minimal number of unique peptides required for protein identification was set to one to meet the special requirements for small protein detection. MaxLFQ-values (Cox et al. 2014) were determined for protein quantification.

For identification of spectra with different spectral libraries, SpectraST (Lam et al. 2007) was used. By application of PeptideProphet (Keller et al. 2002) and ProteinProphet (Nesvizhskii et al. 2003) search results were combined on peptide and protein level and the corresponding FDRs were calculated. Output data were filtered for 1% FDR on spectrum, peptide, and protein level. IonQuant (Yu et al. 2021) was used to determine MaxLFQ-values for protein quantification. At least one ion was required for quantification and match between runs was enabled. Protein identification originating from a single unique peptide were only accepted, if the corresponding MS/MS-spectrum contained at least five consecutive b- or y-ions.

Sample processing for DIA data, acquired to determine the limits of small protein quantification, is described in the Supplemental material.

## Results

### Experimental design

This study was designed to systematically evaluate different MS data processing strategies for their performance in protein identification and protein quantification. Moreover, a special focus was put on small proteins (length ≤100 amino acids) as this challenging class of proteins has been comparatively understudied in eukaryotic and prokaryotic species.

**Figure 1:**
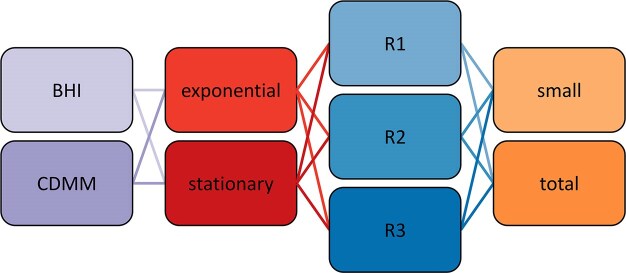
To generate the dataset used in this study *C. difficile* was grown in two media (first column, purple), harvested in two different growth phases (second column, red) with three biological replicates each (third column, blue). The resulting samples were prepared with two different methods (last column, orange) to obtain either the total protein extract or enrich for small proteins.

In order to provide a comprehensive dataset which allows both, robust estimation of method performance and mapping of the so far hidden “small proteome” in *C. difficile*, this anaerobe Gram-positive bacterium was cultured in complex BHI and defined chemical medium and harvested during exponential growth and in stationary phase (Fig. 1). Each sample was generated with three independent biological replicates and further subjected to total protein extraction and enrichment of small proteins prior to MS analyses. For enrichment of small proteins, solid phase extraction was selected as a well-established exemplary method. An overview on alternative methods to enrich small proteins can be found elsewhere (Cassidy et al. 2021).

Obtained mass spectra were identified either by searching them against a SD or against spectral libraries. While application of SDs for processing of mass spectrometric data is still the mostly used method for protein identification and quantification, searches against spectral libraries have proven to result in higher protein identification rates and more robust protein quantification (Lam et al. 2007, Junker et al. 2018b, Fernández-Costa et al. 2020, Hentschker et al. 2020) and are meanwhile standard when analysing mass spectra acquired in DIA mode. In this study, different types of spectral libraries were applied, namely an experimental spectral library (ExSpLib) containing actually acquired mass spectra, a spectral library containing spectra predicted by machine learning (MaLeSpLib), and a merged spectral library (merged SpLib) where spectra predicted by machine learning were added for those peptides, which were not already contained in the ExSpLib. More details on the SD, the generation of the spectral libraries and their sizes can be found in the section “Material and methods” and in Table S2. Following the quality criteria recently reported in the field of peptidomics and small protein identification (Slavoff et al. 2013, D’Lima et al. 2017), protein identification based on one single peptide were only accepted if the corresponding MS/MS-spectrum contained at least five consecutive b- or y-ions. The results obtained from each data processing strategy were examined in terms of number of protein identification (proteins identified with at least one unique peptide in any of the samples), and number of quantified proteins (identified and with an available quantitative value in at least two out of three biological replicates of any condition defined by medium and growth phase). All results were analysed based on total proteins but also with special emphasis on small proteins (Tables S3 and S4). Protein groups, representing <2% of the hits in the dataset, were removed for the analyses as grouping is slightly different in the search algorithms applied during database or spectral library search.

### Importance of sample enrichment for quantification of small proteins

**Figure 2:**
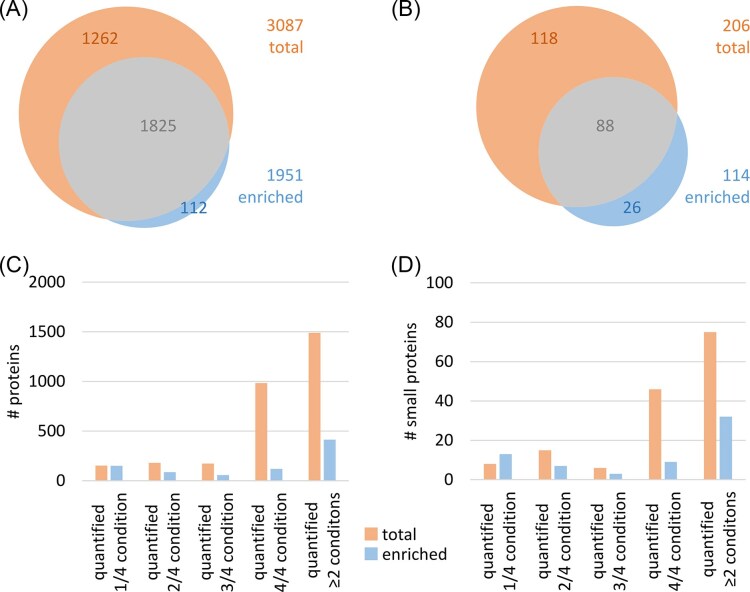
(A) Number of identified proteins after preparation of the total protein sample (orange) or after small protein enrichment by solid phase extraction (blue). (B) Number of identified small proteins (≤100 amino acids) after preparation of the total protein sample (orange) or after small protein enrichment. (C) Number of quantified proteins in total protein extracts (orange) and solid-phase enriched samples (blue). (D) Number of quantified small proteins in total protein extracts (orange) and solid-phase enriched samples (blue). A condition is defined by the used medium (BHI or CDMM) and growth phase (exponential growth or stationary phase) (Fig. 1).

While it is well known that enrichment of small proteins can result in higher identification rates and may also lead to more robust protein identification between replicates (Cassidy et al. 2021, Fabre et al. 2021), this study examined the effect of small protein enrichment on their quantification by processing all samples either as total protein extract or enriched for small proteins using solid phase extraction (Bartel et al. 2020) prior to MS analyses (Fig. 1). As expected, the number of proteins identified by combination of all three identification strategies was reduced after small protein enrichment which is depleting larger proteins from the sample (Fig. 2A). Although enrichment also results in an overall lower number of identified small proteins, 26 small proteins could only be identified after enrichment (Fig. 2B).

Surprisingly, the quantification rate of small proteins after small protein enrichment did not improve compared to that found without enrichment. Still, enrichment for small proteins allows quantification of proteins, which cannot be detected in the total protein extract. Together with previously published results (Cassidy et al. 2019, Bartel et al. 2020, Petruschke et al. 2020) the data suggest that complementary sample preparation methods might be beneficial for both small protein identification and quantification.

### Comparison of protein identification strategies

Utilizing spectral libraries for protein identification has demonstrated increased identification rates and more reliable protein quantification as compared to searches against SDs (Lam et al. 2007, Junker et al. 2018b, Fernández-Costa et al. 2020, Hentschker et al. 2020). Hence, it was tempting to explore the performance of spectral libraries also for quantification of the difficult to analyse group of small proteins. Moreover, a comparison was also made for experimental and predicted spectral libraries as both of them have different advantages and limitations. The dataset of this study (Fig. 1) was therefore processed with different strategies, namely a SD search as well as searches against an experimental spectral library (ExSpLib) and a predicted spectral library (MaLeSpLib) (Table S3). It is important to note that the approach of applying predicted spectral libraries differs from analysis strategies used in software such as DIA-NN and MSBooster, where spectra are predicted on-the-fly and are compared with extracted fragment masses or, in the case of MSBooster, used to rescore initial peptide identifications. This contrasts with our approach, which utilizes precalculated spectral libraries as a complete reference for spectral matching.

**Figure 3:**
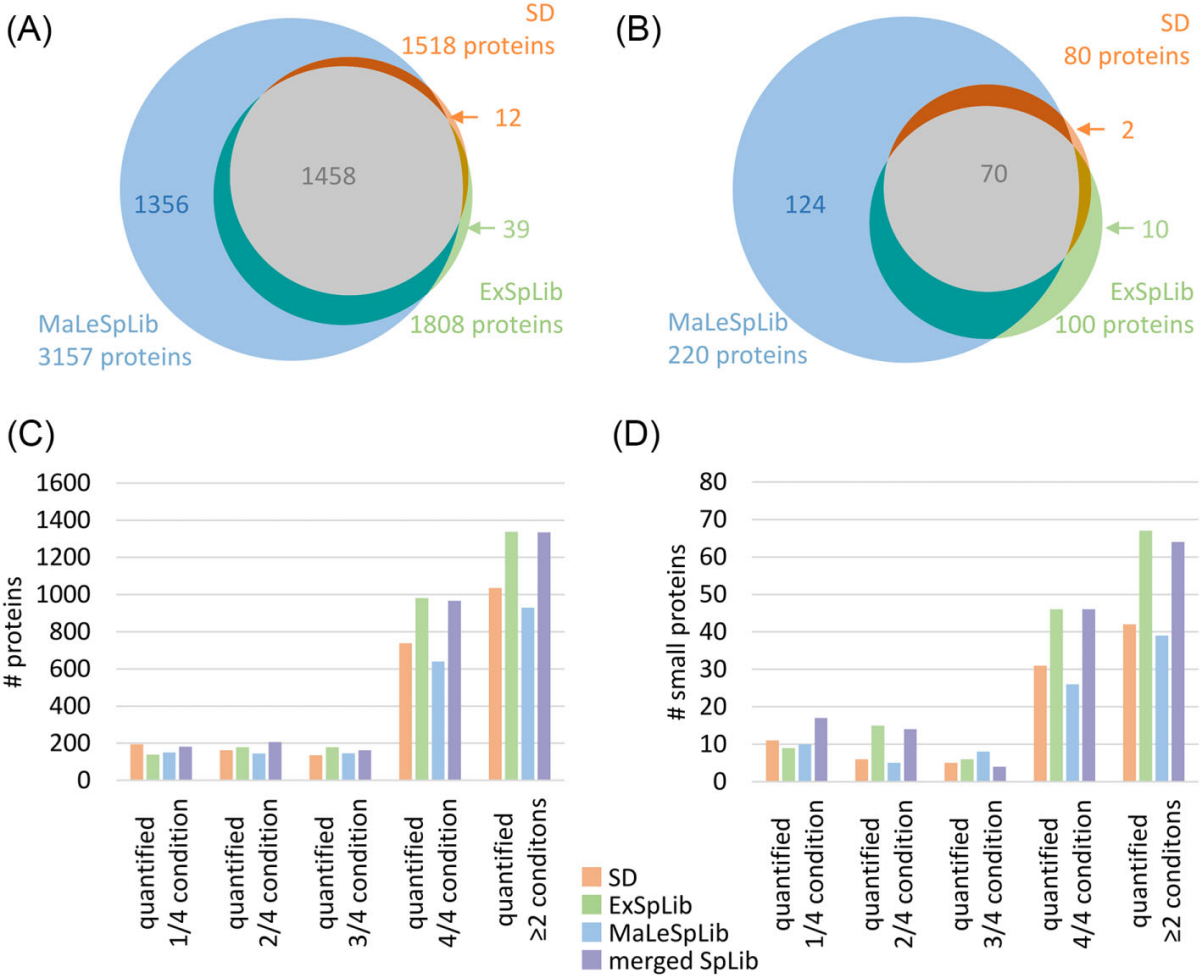
(A) Total number of identified proteins after data processing with a SD (orange), an experimental spectral library (ExSpLib, green), or a predicted spectral library (MaLeSpLib, blue). (B) Number of identified small proteins (≤100 amino acids) after data processing with SD (orange), ExSpLib (green), or MaLeSpLib (blue). (C) Number of quantified proteins after application of different search strategies. (D) Number of quantified small proteins after application of different search strategies. A condition is defined by the used medium (BHI or CDMM) and growth phase (exponential growth or stationary phase).

In concordance with the current knowledge, the number of identified proteins from total protein extracts and small protein -enriched samples depended on the search strategy with higher identification rates for the spectral library approaches and the MaLeSpLib leading to the highest number of identified proteins (Fig. 3A). This was also reflected in the number of identified small proteins (Fig. 3B).

**Figure 4:**
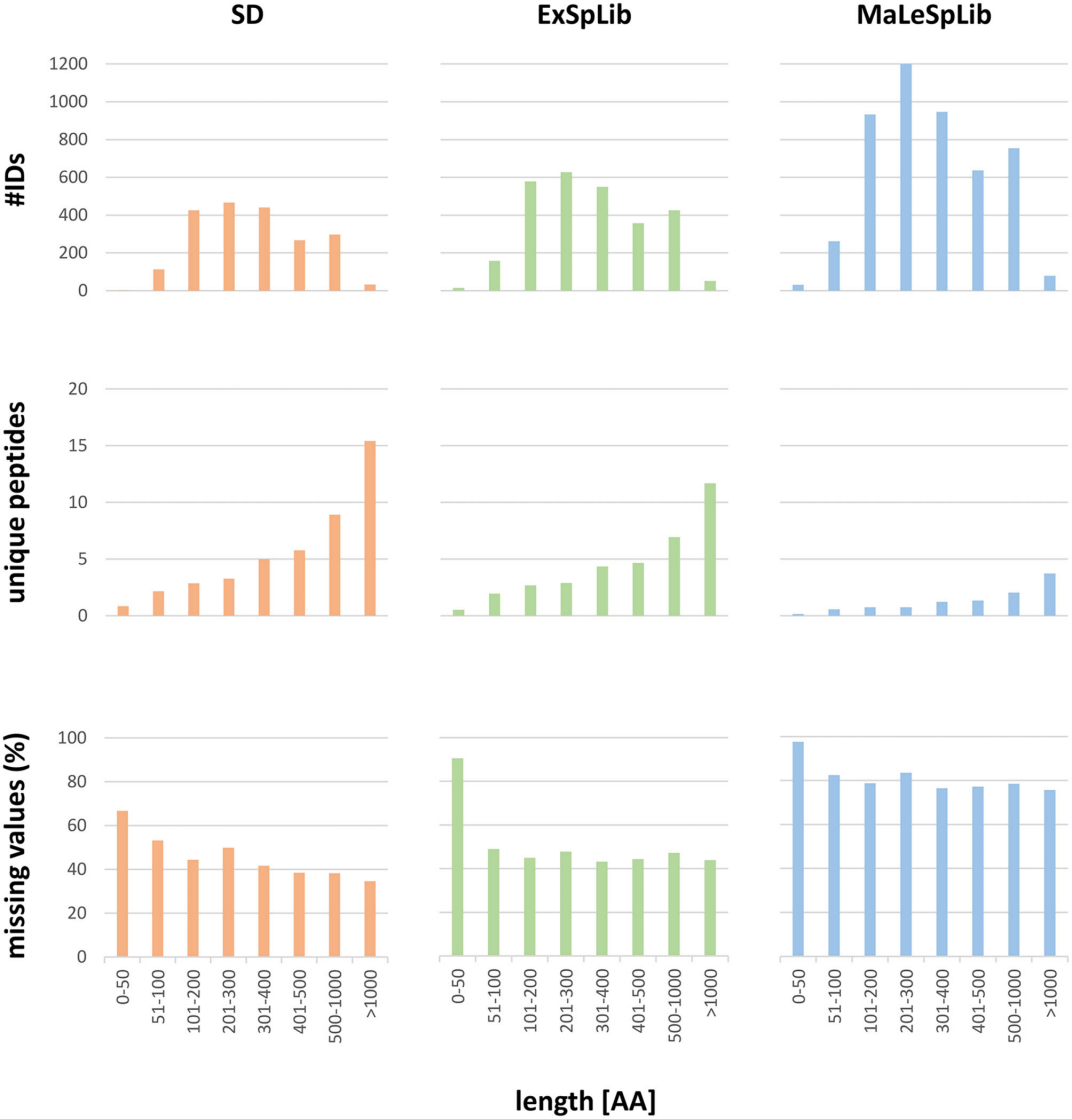
The number of protein identifications, assigned unique peptides per protein, and the fraction of missing values for proteins identified after data processing with a SD (left column, orange), an experimental spectral library (ExSpLib, middle column, green), or a predicted spectral library (MaLeSpLib, right column, blue) was binned for the protein’s length. Detailled data are provided in Table S4.

Of note, the reliable detection of a protein in any of the replicates of a given sample was sufficient to render a protein “identified.” In contrast, the definitions applied in this study consider a protein to be “quantified” if it has been detected in at least two replicates of a given condition. Although the MaLeSpLib performed best in (small) protein identification, protein quantification rates were differentially influenced by the protein identification strategy. Whereas searches against a SD or ExSpLib resulted in high protein quantification rates of ≥80% for all proteins identified, a significantly smaller fraction of proteins could be quantified with MaLeSpLib (34.2%) (Fig. 3C). The effect of the applied protein identification strategy was even more pronounced when small proteins were in the focus of the analyses. Whereas quantification rates for small proteins were 66.3% and 75.3% for searches against SD and ExSpLib, respectively, only 22.2% of the small proteins were quantified with searches against the MaLeSpLib (Fig. 3D). In an attempt to understand the driving factors of these observations in more detail, the number of protein identifications, assigned unique peptides, and the fraction of missing values were binned for protein sizes (Fig. 4, Table S4).

Although the application of the MaLeSpLib allowed for a high number of identified proteins over all size bins as spectra for more peptide sequences are contained in this library, the number of assigned unique peptides for these proteins is significantly lower than for those proteins identified with SD or ExSpLib (Fig. 4). Indeed, the spectra quality in the MaLeSpLib is significantly different from those contained in the ExSpLib (Fig. S1). Ultimately, this leads to occasional peptide identification associated with a high number of missing values in the MaLeSpLib dataset, which results in a comparable low fraction of quantified proteins with this search approach (Fig. 3C and D). Additionally, in order to be able to compare the quantification performances of the different search approaches, this study employed the IonQuant algorithm (Yu et al. 2021) to determine LFQ values from spectral library searches. However, successful peak integration on MS1 levels requires multiple detection of the precursor ion mass in the analytical window of the MS-measurements rendering determination of LFQ values especially challenging for low abundant peptides. We hypothesize that these effects would have been less pronounced if quantification methods based on spectral counting would have been applied.

In order to potentially combine the advantages of ExSpLib and MaLeSpLib, both types of spectral libraries were merged, whereby experimentally acquired spectra were supplemented with predicted spectra of peptide ions not yet included in the ExSpLib. Interestingly, application of the merged SpLib yielded only slightly lower quantification rates than ExSpLib (Fig. 3C and D). In detail, quantification rates did not drop to the same extend as observed after processing with the MaLeSpLib, which demonstrates that this reduction is not mainly rooted in the vastly increased search space.

### Limits of small protein quantification

To determine the limits of small protein quantification with the different workflows, a protein sample obtained from exponential growing *C. difficile* in BHI was spiked in technical triplicates with six purified small proteins from *H. volcanii* H119 ranging from 38 to 78 amino acids in length (HVO_0758, HVO_2212, HVO_2753, HVO_2922, HVO_2983, and HVO_A0101) (Supplemental material and Table S2). Spike-in concentrations were selected to cover the range of low- and medium-abundant proteins in a background proteome, hence ranging from 0.5 to 500 pg of spike-in protein per µg of *C. difficile* proteins. All samples were subjected to total protein preparation or enrichment of small proteins by solid-phase extraction prior to MS analyses. Identification of mass spectra was achieved by SD search or by application of an experimental spectral library (ExSpLib), or a predicted spectral library (MaLeSpLib).

Whereas HVO_0758, HVO_2212, and HVO_2922 (56, 78, and 60 amino acids long, respectively) were quantified frequently (Table 1), HVO_2983 (38 AA) could not be identified in any of the samples. HVO_A0101 (61 AA) and HVO_2753 (62 AA) were only identified in one sample and were therefore not considered to be robustly quantified in this study.

**Table 1:**
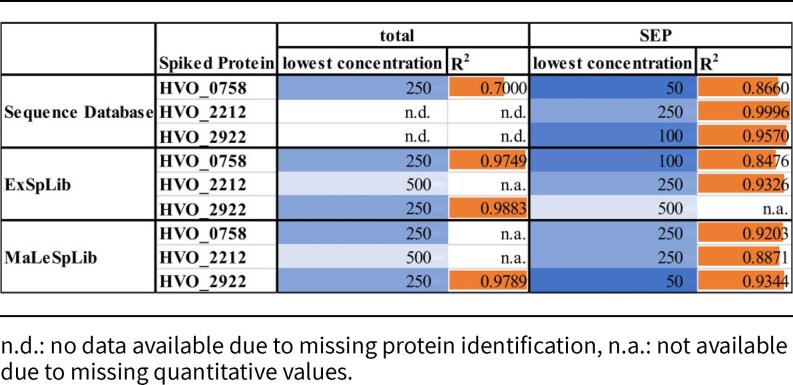
Quantification limits of small proteins in a bacterial cell lysate. Data were obtained either for total protein extracts (total) or after enrichment of small proteins (SEP). Data were derived from triplicate MS/MS experiments in DDA mode. Identification of mass spectra was achieved by SD search or by application of an experimental spectral library (ExSpLib) or a predicted spectral library (MaLeSpLib). The lowest concentration, in which a protein could be quantified (providing a quantitative value in at least 2 of 3 technical replicates) is given in pg per µg background proteome and colored in blue where darker color shades indicate a lower limit of detection. The coefficient of determination (*R*^2^) for the correlation of protein concentration to peak area is represented by orange bars with longer bars indicating higher *R*^2^.

A higher number of small proteins could be quantified and their limit of detection was lower when samples were specifically enriched for small proteins (Table 1). Together with the fact, that small protein enrichment gave rise to proteins not yet detected in the nonenriched sample (see the section “Importance of sample enrichment for quantification of small proteins”), this emphasizes the benefits of sample enrichment for robust quantification of small proteins. Moreover, the observed quantitative values showed good linearity for the three frequently quantified proteins, demonstrating that the additional processing steps for small protein enrichment do not introduce biases in the comparison of their abundances (Table 1).

When comparing different search strategies in the context of quantification limits, it becomes evident that the application of spectral libraries can enhance the number of quantifiable small proteins by providing confident identification of their peptides in a given dataset, especially when no enrichment of small proteins was carried out (Table 1). This is in line with the data obtained from the comparison of different search strategies (Fig. 3C and D).

Of note, the benefit of enrichment before small protein quantification was also significant, when samples were analysed in DIA mode (Table S5). Moreover, also the number of quantifiable small proteins is enhanced when samples are acquired in DIA mode compared to DDA. More detailed results on the limits of small protein quantification in DIA mode can be found in the Supplemental material.

### The extended (small) proteome of *C. difficile*

In this study, a comprehensive dataset was generated (Fig. 1), which was processed by searching acquired mass spectra either against a SD or against different spectral libraries, namely the ExSpLib, MaLeSpLib, and a merged SpLib obtained from the latter. Appending all search results of this study on the level of identified proteins resulted in 3202 protein hits of which 1840 proteins have been identified independently with at least two of the search approaches. As 3781 proteins have been predicted by the most recent genome analyses (Dannheim et al. 2017) this represents an 84.7% coverage of the predicted proteome of *C. difficile* 630 Δ*erm*. Due to the lower sensitivity and the restricted search space for SD and ExSpLib as compared to the MaLeSpLib, respectively, the identification rate in the MaLeSpLib was much larger (Fig. 3A), resulting in a proteome coverage of 48.7% if only these proteins are considered, which have been identified with at least two search approaches. Therewith, the dataset presented here is, to our knowledge, the most comprehensive protein repository for *C. difficile* 630 Δ*erm* to date (Table S1).

The dataset processed with the ExSpLib contained the highest number of robustly quantified proteins (quantified in at least two out of three biological replicates of any condition defined by medium and growth phase). Hence, the ExSpLib derived data for 1459 proteins were used as base for a quantitative analyses of protein abundance of which 1308 proteins (Table S6) could be robustly quantified in at least two of the conditions enabling analyses of differential protein abundance. Indeed, 1213 of these proteins have also been reported earlier in Otto et al. (2016), who compared protein abundances of *C. difficile* 630 Δ*erm* grown in the same media as used in this study. Whereas quantitative data of both studies were consistent, we herewith add information on differential protein abundance for 95 more proteins including six small proteins with up to 100 amino acid length. Additionally, this dataset adds information on changes in protein abundance in two different growth phases (exponential and stationary phase). Another recently published dataset also compares proteins extracted from growing and nongrowing cells in BHI and minimal medium (Trautwein-Schult et al. 2018). Filtering this dataset for statistical significance (two-way ANOVA, p<0.01) and abundance changes of at least 1.74-fold (corresponding to log2FC>|0.8|) revealed only 56 differently abundant proteins after metabolic labeling. In contrast, the current study reports 363 differentially abundant proteins thereby adding robust quantitative information with less missing values to the current knowledge.

**Figure 5:**
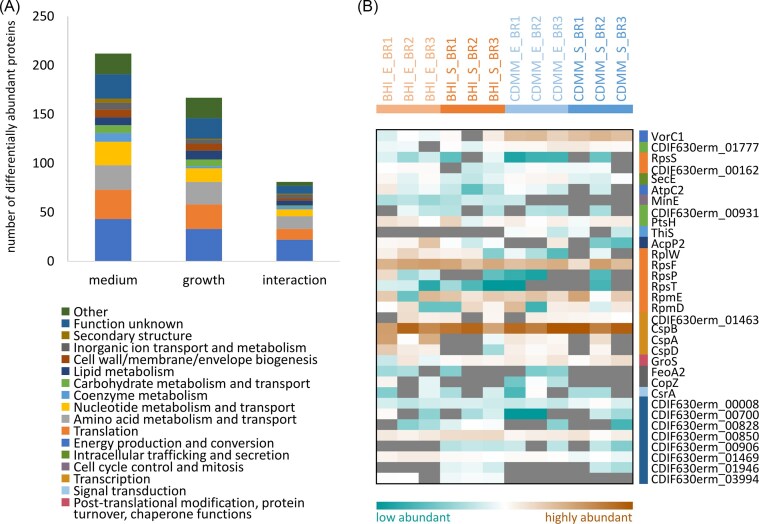
(A) Functional categorization of differentially abundant proteins (p<0.01, log2FC>|0.8|) when comparing growth of *C. difficile* 630 Δ*erm* in complex BHI or defined chemical medium (medium), in exponential versus stationary phase (growth) or by both factors (interaction). Functional categorization is based on COG categories (Galperin et al. 2025). (B) Heat map representing small proteins (≤100 amino acids), which have been robustly quantified (quantitative value in at least two out of three biological replicates [BR1-3]) in at least two of the examined conditions (defined as the combination of medium [BHI: complex medium, CDMM: minimal medium] and growth phase [E: exponential, S: stationary). Heat map tiles are colored according to the protein abundance represented by the MaxLFQ value. The color in front of the protein names represents the functional categorization with the same colors used as in panel A. The first five lines of the heat map represent small proteins with differential abundance (p<0.01, log2FC>|0.8|).

Most of these 363 proteins (212) show differential protein abundance caused by the different media (Fig. 5A), whereas 167 proteins were altered dependent on the growth phase and for 81 proteins the medium influenced the differential abundance in the growth phase (termed “interaction” in Fig. 5A). In each of these groups, the highest number of proteins function in energy production and conversion, translation, and amino acid metabolism and transport. The higher expression of enzymes involved in vitamin and purine biosynthesis and the reduced abundance of proteins involved in butanoate fermentation in minimal medium reported by Otto et al. (2016) as well as the observed comparable abundance of proteins with functions in DNA metabolism, protein synthesis, and the cell envelope in the different media is also detectable in the dataset of this study.

In this study, we present first-time evidence for the actual translation of 639 proteins, out of which 82 represent small proteins with up to 100 amino acid length. Additionally, the so far hidden “small proteome” of *C. difficile* 630 Δ*erm* could be extended to 215 proteins out of which 100 could be identified with at least two search approaches. Considering the 350 predicted small proteins for this organism (Dannheim et al. 2017), the coverage of the small proteome is 61.4% (28.6% if identification by at least two search approaches were required). Data processing with the ExSpLib allowed robust quantification of 46 small proteins, of which 33 were quantified in at least two conditions and can thus be used for differential abundance analyses (Fig. 5B). Given the poor characterization of many small proteins, it is not surprising that for a considerable fraction of the quantified small proteins (8) no function can be predicted. Out of the remaining 25 small proteins 32% (8) are small ribosomal proteins fulfilling functions during translation and another 16% (4) are represented by cold shock proteins categorized in the group “transcription.” Only five small proteins show a differential abundance (p<0.01, log2FC>|0.8|) in this study. In line with the current knowledge, the small ribosomal proteins RpsS was depleted in stationary phase compared to exponential growth. In contrast, the L14E/L6E/L27E-like ribosomal protein CDIF630erm_00162 was higher abundant during exponential growth in BHI compared to stationary phase in the same medium suggesting an adaption of the ribosomal protein complex in these conditions. The translocase SecE shows a similar pattern with higher abundance during exponential growth than in stationary phase in both of the media pointing at a reorganization of the Sec-translocon during transition from growing to nongrowing conditions. The subunit VorC1 of the 3-methyl-2-oxobutanoate dehydrogenase functioning in energy production and conversion as well as CDIF630erm_01777, the IIB component of a lactose/cellobiose-family PTS system, showed medium-dependent differential abundance with enhanced protein amounts when cells have been grown in minimal medium. This is most probably linked to different nutrient availability in the two media.

## Discussion

In this study, different sample preparation methods and data processing strategies have been evaluated for (small) protein identification and quantification. Moreover, MS evidence for the translation of 639 proteins in *C. difficile* 630 Δ*erm* is provided for the first time and 61.4% of the “small proteome” of this important human pathogen could be mapped.

Although it is well known that small protein identification can benefit from dedicated sample preparation methods (reviewed in Cassidy et al. 2021), the effects on robust quantification of this challenging class of proteins was not yet studied systematically. Although in this study, the quantification rate of small proteins did not improve following their enrichment, it enabled the quantification of proteins that are undetectable in the total protein extract. Moreover, their enrichment did improve the quantification limit for the spiked small proteins. Still, unlike published earlier (Bartel et al. 2020), not only fewer proteins were identified after enrichment (Fig. 2A and B), but also fewer peptides/ions/spectra per proteins were detected independent of protein size. This effect might be due to different digestion methods used in Bartel et al. (2020, in solution digest) and in this study (S-Trap digest). Although it has been reported multiple times, that S-Trap digests result in efficient protein digestion, high numbers of protein identifications, as well as sensitive and reproducible protein quantification (Ludwig et al. 2018, Antelo-Varela et al. 2019), the application of this protocol on small protein enriched samples seems to cause significant protein loss. In any case, according to the current knowledge (Ma et al. 2016, Cardon et al. 2020, Petruschke et al. 2020, Wang et al. 2021), the heterogeneity of small proteins in terms of their physicochemical properties would most probably require the combination of multiple complementary methodologies to improve both the number of identified small proteins in a sample as well as their robust quantification in biological datasets.

Besides the application of multiple sample preparation methods, subcellular fractionation of bacterial cell samples might support an enhanced (small) proteome coverage. However, a higher degree of sample fractionation is usually accompanied by the requirement for higher amounts of sample material. According to PSORTb (Yu et al. 2010) 54.6% of the 3781 annotated *C. difficile* proteins are predicted to be located in the cytoplasm whereas only 26.1% and 1.1% of the proteins are assigned to the membrane (including cell wall proteins) and extracellular fraction, respectively. This already implies that a higher sample volume is necessary to prepare the same amount of cytosolic, membrane, and secreted (small) proteins. Indeed, in *Bacillus subtilis*, which has a comparable distribution of subcellular protein localization than *C. difficile*, 300 times more cell culture needs to be harvested in stationary phase to prepare the same amount of extracellular proteins than compared to cytosolic proteins. If extracellular proteins are harvested at timepoints with less active secretion and/or cell lyses, even a 1000 times higher culture volume is necessary (F. Grilli, personal communication). These considerations multiply with the fact, that enrichment of small proteins by solid phase extraction usually starts with 50 times more starting material than needed for a nonenriched sample. However, even without subcellular fractionation this study was able to report 3202 identified proteins. Assuming 3256 protein-coding genes that are expressed during growth in BHI in late-exponential phase (Lamm-Schmidt et al. 2021), this study almost covers the complete potential proteome.

If, like in this study, multiple data processing approaches are used to enhance the number of protein identifications and thus to achieve a more robust protein quantification, this raises the question whether all hits provided by different algorithms are valid. Indeed, it has already been reported that diverse spectrum preprocessing and scoring functions in different search algorithms lead to marginally different sets of reported peptides (Searle et al. 2008). In the current dataset, 346 135 spectra could be assigned to *C. difficile* proteins out of which 201 422 (58.2%) were identified by at least two of the applied search approaches. Only 18 022 spectra (8.9% of the spectra identified by multiple search strategies) were assigned to different proteins by the different search approaches.

To compare protein abundances of (small) proteins in an unbiased manner, robust datasets with the least number of missing values are anticipated. The systematic evaluation of proteomic workflows in this study has shown that enrichment for small proteins can reduce the limit of detection for this challenging protein class. Moreover, the application of spectral libraries for data processing did not only result in a lower limit of detection of small proteins (Table S5) but also enhanced the number of robustly quantified proteins (Fig. 3D). In order to generate quantitative datasets with an even higher number of robustly quantified proteins, DIA approaches (Ludwig et al. 2018) might be a valuable option. DIA workflows have shown to exhibit higher reproducibility and proteomic depth compared to data-dependent acquisition (DDA) methods (Bruderer et al. 2017, Collins et al. 2017, Vowinckel et al. 2018). Indeed, here we found that the number of quantifiable small proteins is enhanced when samples are acquired in DIA mode compared to DDA (Table S6).

In this study, 215 small proteins (length ≤100 amino acids) of *C. difficile*, representing 61.4% of the predicted “small proteome”, could be detected by MS. Also other recent studies, which focus on the identification of small proteins, report a high number of small proteins but were only able to cover 34.6%, 27.6%, and 7.6% of the predicted small proteins in their model organisms *B. subtilis* (Bartel et al. 2020), *H. volcanii* (Hadjeras et al. 2023a), and *Sinorhizobium meliloti* (Hadjeras et al. 2023b), respectively, by mass-spectrometry alone. Therewith the current study represents one of the most comprehensive “small proteomes”, which is most likely attributed to the combination of sample preparation methods and broad application of data processing approaches.

This study is currently limited to the identification of already annotated open reading frames. In order to facilitate the discovery of not-yet annotated small proteins, proteogenomic approaches need to be applied. Such proteogenomic tools integrate genomics and proteomics to identify previously unannotated proteins with the aim to enhance or refine genome annotations (Nesvizhskii 2014). Using a six-frame translation-based protein database, Slavoff et al. (2013) successfully detected 86 novel small proteins in a human cell line. The application of integrated proteogenomics search databases (iPtgxDB) allowed for identification of 22, 11, and 3 novel small proteins in *Bartonella henselae* (Omasits et al. 2017), *S. meliloti* (Hadjeras et al. 2023b), and *B. subtilis* (Bartel et al. 2020), respectively. However, databases used in proteogenomic approaches are usually huge which complicates prediction of spectra from these databases by machine learning. The required computational power results in long processing times to generate corresponding spectral libraries. Although the application of resulting spectral libraries is not more time demanding than other search approaches, the time needed to generate the spectral libraries might only pay of if the spectral library is used for many and/or very comprehensive experiments.

## Supplementary Material

uqag002_Supplemental_Files

## Data Availability

The mass spectrometry proteomics data have been deposited to the ProteomeXchange Consortium (http://proteomecentral.proteomexchange.org) via the PRIDE partner repository (Perez-Riverol et al. 2022) with the dataset identifier PXD065346.
