# Supplementary material for: Less missing values—evaluation of proteomics workflows for the quantification of (small) proteins
Source: Microlife. 2026 Jan 10;7:uqag002. doi: 10.1093/femsml/uqag002 (PMC12850539; doi:10.1093/femsml/uqag002)
Supplement: uqag002_Supplemental_Files [file uqag002_supplemental_files.zip › SupplementalMaterial.docx]

**Supplemental Material**

Less missing values – evaluation of proteomics workflows for the quantification of (small) proteins

Jürgen Bartel^1^, Vaikhari Kale^1^, Dennis Joshua Pyper^2^, Harald Schwalbe^2^, Sandra Maaß^1^*

^1^ University of Greifswald, Institute of Microbiology, Department of Microbial Proteomics, Greifswald, Germany

^2^ Goethe University Frankfurt, Institute for Organic Chemistry and Chemical Biology, Center of Biomolecular Magnetic Resonance (BMRZ), Frankfurt am Main, Germany

Running title: Optimized quantification of (small) proteins

Keywords: mass spectrometry, peptidomics, *Clostridioides difficile*, SEPs, sProteins, database search, spectral library

*Correspondence: S.M. (Phone: +49 3834 4205921, Fax: +49 3834 4205909, Email: sandra.maass@uni-greifswald.de)

## Supplemental Methods

### Expression and purification of standard proteins for evaluation of protein quantification

The ORFs of five small proteins from *Haloferax volcanii* ranging from 38 to 78 amino acids (HVO_0758, HVO_2212, HVO_2983, HVO_2922, and HVO_A0101) were introduced into a pE-SUMO vector, adding a SUMO fusion protein and a His_6_-tag to the N-terminus of the proteins. HVO_2753 ORF (59 amino acids) was placed into a pGEX-CS vector which adds a GST-tag for purification to the N-terminus. The plasmids were used to transform *E. coli* T7 Express cells and small-scale expressions were carried out in 100 mL of LB medium with 1 mM of ampicillin added. All cultures were grown at 37 °C until OD_600_ was between 0.6 and 0.7. Protein overexpression was subsequently induced by adding 1 mM IPTG and cultures were incubated overnight at 18 °C. Cells were then harvested by centrifugation at 6,200 xg for 15 min at 4 °C. The cell pellets were resuspended in Buffer A (see Supplemental Table S2 for protein-specific buffer composition) together with protease inhibitor (cOmplete, Roche) and lysed by french press. The lysate was then cleared by centrifugation for 40 min at 40,000 xg and 4 °C.

For proteins carrying a SUMO and His_6_-tag, the cleared lysate was loaded onto a NiNTA column and for the GST-tagged protein onto a GST column. Elution was then accomplished with a gradient of buffer B (Supplemental Table S2) to 500 mM imidazole or to 10 mM reduced glutathione, respectively. Cleavage of the affinity tags was carried out by addition of Ulp1 to the SUMO-tagged proteins or TEV protease to the GST-tagged protein and subsequent incubation overnight at 4 °C. The SUMO-tagged proteins were then loaded onto a NiNTA column again for removal of the SUMO-tag from the solution. The proteins of interest were collected in the flow-through. As a last purification step, size-exclusion chromatography was carried out for all proteins to obtain >95% pure protein (see Supplemental Table S2 for used buffers).

Protein concentration of the individual purified small proteins was determined by their UV absorption at 280 nm using molar absorption coefficients calculated by ExPASy’s ProtParam tool ^1^

### Generation of HCD-type spectral libraries

The experimental spectral library (ExSpLib) used to search the HCD-spectra of the DIA-experiment (see below) was created as described in the main text for the CID library expect that data for generation of the ExSpLib were search with MS-Fragger as this search engine has shown to be especially suited for HCD data.

Machine learning spectral libraries (MaLeSpLib) were generated as described in the main text. Predicted spectra were based on the sequence database for *C. difficile* 630∆*erm* ^2^ supplemented with 116 common laboratory contaminants and, for determination of the limits of small protein quantification, also with the protein sequences of the six proteins from *Haloferax volcanii* that were spiked to the sample. The spectra contained in the MaLeSpLib used to search the HCD-spectra of the DIA-experiment (see below) were predicted with the Prosit-HCD2020 model.

### Mass spectrometry in DIA mode

LC-MS/MS analyses of synthetic peptides in DIA mode were performed with an EASY-nLC 1000 liquid chromatography system coupled to a Q Exactive mass spectrometer (Thermo Fisher Scientific, USA). The LC was equipped with a self-packed analytical column (OD 360 µm, ID 75µm, length 20 cm) filled with 1.9 µm diameter C18 particles (Dr. Maisch HPLC GmbH). Peptides were eluted using a binary non-linear gradient of 5-99% acetonitrile in 0.1% acetic acid over 161 min at a flow rate of 300 nl/min, and subjected to electrospray ionization-based MS. Each full scan in the Orbitrap with a resolution of 70,000 was followed by HCD fragmentation and 23 DIA scans with variable-width windows covering the mass range between 320 and 1850 m/z at a resolution of 35,000. The cycle time was 3.7 s, ensuring at least 8 scans over the width of a typical liquid chromatography peak (30 s).

### Data processing of DIA data

Raw data from DIA runs were processed with DIA-NN (v1.8.1) ^3^ using an internally generated deep-learning in-silico spectral library based on the sequence database described in the main manuscript (default workflow), the HCD-type ExpSpLib or the HCD-MaLeSpLib. The latter two libraries were converted into DIA-NN’s own *.tsv* format and retention times for all peptides were predicted using the deeplc_hela_hf model from koina ^4^ prior to searching. Tryptic digestion with up to two missed cleavage sites, peptide length between 7 and 35 amino acids, precursor mass-to-charge ratio between 300 and 1800 Th, precursor charge state between +1 and +4, fragment mass-to-charge ratio between 200 and 2000 Th, fixed carbamidomethylation at cysteine residues and optional oxidation of up to three methionine residues per peptide were considered. Mass accuracy was set to 10 and 20 ppm for MS1 and MS2 scans respectively, peak-window width was fixed at 15 scans and the neuronal network was trained for two passes. The quantification strategy was set to “robust LC (high-accuracy)” and the match between runs option was activated. The main report was filtered to 1% precursor-FDR by DIA-NN and further filtered in R (v4.2.2) for a Lib.Q.Value ≤ 0.01 and Quantity.Quality ≥ 0.5. Using diann-rpackage, Max-LFQ values based on normalized precursor intensity (Precursor.Normalised) were calculated for proteins with at least 2 unique or razor peptides and protein Max-LFQ values were reported when a protein was quantified in at least two technical replicates of a sample.

## Supplemental Results

### Comparison of protein identification strategies


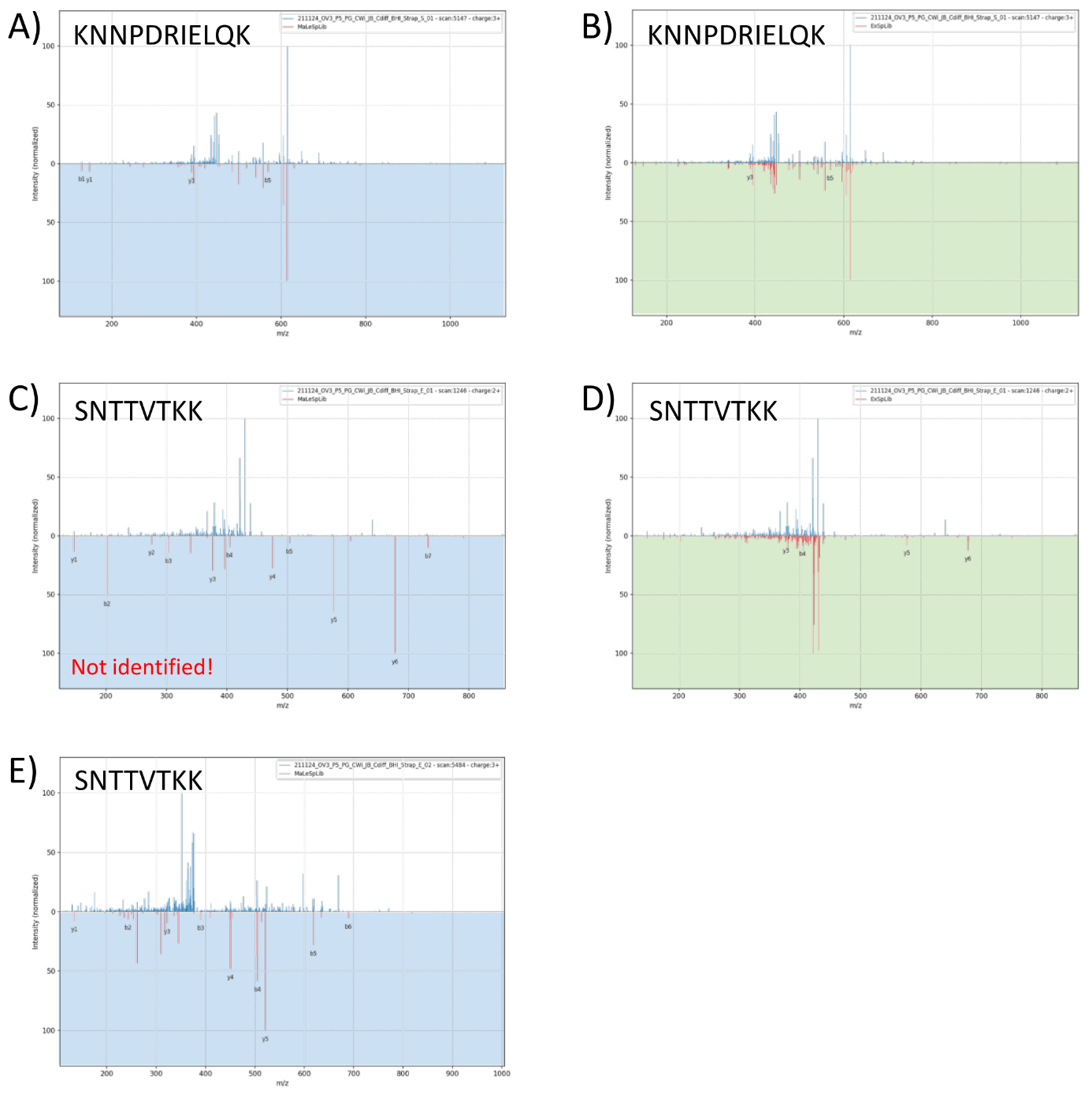


Supplemental Figure S1: Selected spectra exemplifying the match between acquired spectrum (top of each panel) to the spectrum stored in the spectral library (bottom of each panel). Library spectra stored in the MaLeSpLib are highlighted blue, spectra contained in the ExSpLib are highlighted in green. Selected examples originate from peptides matching to proteins with a size smaller than 50 amino acids. The peptide in the the first example (A, B) was identifed with both, the MaLeSplib (A) and the ExSpLib (B). In the second example (C, D) the peptide was identified only with the ExSpLib (D), which contained a spectrum which was significantly different from that contained in the MaLeSpLib (C). The third peptide (E) could only be identified with the MaLeSpLib as it was not contained in the ExSpLib.

### Limits of small protein quantification in DIA mode

To determine the limits of small protein quantification with the different workflows, a protein sample obtained from exponential growing *C. difficile* in BHI was spiked in technical triplicates with six purified small proteins from *Haloferax volcanii* H119 ranging from 38 to 78 amino acids in length (HVO_0758, HVO_2212, HVO_2753, HVO_2922, HVO_2983, and HVO_A0101) (Supplemental Table S2). Spike-in concentrations were selected to cover the range of low- and medium-abundant proteins in a background proteome hence ranging from 0.5-500 pg of spike-in protein per µg of *C. difficile* proteins. All samples were subjected to unbiased sample preparation or enrichment of small proteins by solid phase extraction prior to MS analyses. Fragmentation of peptide ions for MS/MS experiments was performed by HCD in DIA mode. Identification of mass spectra was achieved by application of the default workflow based on a sequence database or by application of an experimental spectral library (ExSpLib), or a predicted spectral library (MaLeSpLib).

Whereas HVO_0758, HVO_2212, and HVO_2922 (56, 78, and 60 amino acids long, respectively) were quantified frequently (Supplemental Table S5), HVO_2983 (38 AA) and HVO_A0101 (61 AA) could not be identified in any of the samples. HVO_2753 (62 AA) was not identified when samples were processed with the predicted spectral library. The lower limit of detection and improved coefficient of determination (R^2^) shown for solid phase enriched small proteins in Supplemental Table S5 support the findings from DDA-experiments (Table 1 in main text) that quantification of small proteins can be improved by enrichment of this challenging protein class. However, as also the default workflow for processing of DIA-data relies on a spectral library, which is generated by the processing software based on the provided sequence database, there is no clear advantage of using dedicated spectral libraries, such as an experimental spectral library (ExSpLib), or an externally predicted spectral library (MaLeSpLib) for small protein quantification. Of note, R^2^ are slightly improved when an ExSpLib is applied and the application of MaLeSpLib yielded an identified protein less.

Supplemental Table S5: Quantification limits of small proteins in a bacterial cell lysate. Data were obtained either for total protein extracts (total) or after enrichment of small proteins by solid phase extraction (SEP). Data were derived from triplicate MS/MS experiments in DIA mode. Identification of mass spectra was achieved by application of the default workflow based on a sequence database or by application of an experimental spectral library (ExSpLib), or a predicted spectral library (MaLeSpLib). The lowest concentration, in which a protein could be quantified (providing a quantitative value in at least 2 of 3 technical replicates) is given in pg per µg background proteome along with the coefficient of determination (R^2^) for the correlation of protein concentration to peak area.

|  | | **Default Workflow** | | | | **ExSpLib** | | | | **MaLeSpLib** | | |
| --- | --- | --- | --- | --- | --- | --- | --- | --- | --- | --- | --- | --- |
| **Spiked protein** | | **HVO_ 0758** | **HVO_ 2212** | **HVO_ 2753** | **HVO_ 2922** | **HVO_ 0758** | **HVO_ 2212** | **HVO_ 2753** | **HVO_ 2922** | **HVO_ 0758** | **HVO_ 2212** | **HVO_ 2922** |
| total | lowest concentration | 100 | 250 | 250 | 100 | 100 | 250 | 250 | 250 | 100 | 250 | 250 |
|  | R^2^ | 0.7445 | <0.7000 | <0.7000 | 0.9805 | 0.7651 | 0.9985 | <0.7000 | 0.8201 | 0.7485 | 0.9988 | <0.7000 |
| SEP | lowest concentration | 10 | 50 | 100 | 50 | 10 | 50 | 100 | 100 | 50 | 50 | 50 |
|  | R^2^ | 0.9390 | 0.9804 | <0.7000 | 0.9672 | 0.9622 | 0.9703 | <0.7000 | 0.9433 | 0.9303 | 0.9747 | 0.8796 |

## Supplemental References

(1) Gasteiger, E.; Hoogland, C.; Gattiker, A.; Duvaud, S.; Wilkins, M. R.; Appel, R. D.; Bairoch, A. Protein Identification and Analysis Tools on the ExPASy Server. In *The Proteomics Protocols Handbook*; Walker, J. M., Ed.; Springer Protocols Handbooks; Humana Press: Totowa, NJ, 2005; pp 571–607. https://doi.org/10.1385/1-59259-890-0:571.

(2) Dannheim, H.; Riedel, T.; Neumann-Schaal, M.; Bunk, B.; Schober, I.; Spröer, C.; Chibani, C. M.; Gronow, S.; Liesegang, H.; Overmann, J.; Schomburg, D. Manual Curation and Reannotation of the Genomes of *Clostridium difficile* 630Δ*erm* and *Clostridium difficile* 630. *J. Med. Microbiol.* **2017**, *66*, 286–293. https://doi.org/10.1099/jmm.0.000427.

(3) Demichev, V.; Messner, C. B.; Vernardis, S. I.; Lilley, K. S.; Ralser, M. DIA-NN: Neural Networks and Interference Correction Enable Deep Proteome Coverage in High Throughput. *Nat Methods* **2020**, *17* (1), 41–44. https://doi.org/10.1038/s41592-019-0638-x.

(4) Bouwmeester, R.; Gabriels, R.; Hulstaert, N.; Martens, L.; Degroeve, S. DeepLC Can Predict Retention Times for Peptides That Carry As-yet Unseen Modifications. *Nat Methods* **2021**, *18* (11), 1363–1369. https://doi.org/10.1038/s41592-021-01301-5.
